# Effects of X-ray irradiation on methylation levels of p16, MGMT and APC genes in sporadic colorectal carcinoma and corresponding normal colonic mucosa

**DOI:** 10.1186/1897-4287-10-S3-A12

**Published:** 2012-04-20

**Authors:** Łukasz Krakowczyk, Joanna K Strzelczyk, Sławomir Blamek, Adam Maciejewski, Stanisław Półtorak, Andrzej Wiczkowski

**Affiliations:** 1 Maria Sklodowska-Curie Memorial Cancer Center and Institute of Oncology, Gliwice Branch Department of Oncological and Reconstructive Surgery, Poland; 2 Maria Sklodowska-Curie Memorial Cancer Center and Institute of Oncology, Gliwice Branch Department of Radiotherapy, Poland; 3 Medical University of Silesia in Katowice, Department of General Biology, Zabrze, Poland

## 

Colorectal cancer, one of the most aggressive cancers, occurs with a high incidence in all over the world. Cancer development and progression is dictated by series of alterations in a number genes such as tumor suppressor genes, DNA repair genes, oncogenes and others. DNA methylation is an epigenetic modification that is profoundly altered in most cancers. Radiation is a well-known genotoxic agent and human carcinogen that gives rise to a variety of long-term effects. The inheritance of genomic instability suggests the possible involvement of epigenetic mechanisms, such as changes of methylation of the cytosine residues located within CpG dinucleotides. In the current study we evaluated the effect of X-ray irridiation on methylation levels of p16, MGMT and APC genes in colorectal cancers and normal colonic mucosae. Fresh tissue samples were obtained from 26 patients (age of 26 to 81 years) with primary colorectal adenocarcinoma and corresponding normal tissues. We used methylation-specific polymerase chain reaction (MSP) for analysis of the methylation status of p16, MGMT and APC1A, APC1B genes. Methylation of p16, MGMT, APC1A and APC1B was detected in 42%, 67%, 42% and 20% of tumors before X-ray irradiation and in 63%, 56%, 25% and 31% after radiotherapy, respectively. In corresponding normal colonic mucosa methylation of p16, MGMT and APC1A was detected in 17%, 25% and 8% before and in 19%, 31% and 6% after irradiation, respectively. Differences between groups, although noticeable did not however reach the level of statistical significance.

